# Comorbidity and Pattern of Substance Use in Hospitalized Psychiatric Patients

**DOI:** 10.5812/ircmj.19282

**Published:** 2014-08-05

**Authors:** Zahra Sepehrmanesh, Afshin Ahmadvand, Alireza Moraveji

**Affiliations:** 1 Department of Psychiatry, Kashan University of Medical Sciences, Kashan, IR Iran; 2 Department of Epidemiology, Kashan University of Medical Sciences, Kashan, IR Iran

**Keywords:** Mental Disorders, Comorbidity, Substance-Related Disorders

## Abstract

**Background:**

Substance use in patients with psychiatric disorder is an every-day seen. Detection of this comorbidity can significantly affect the treatment of these disorders, as well as substance use.

**Objectives:**

This study has been conducted to determine the prevalence and pattern of substance use in hospitalized psychiatric patients.

**Patients and Methods:**

In this cross-sectional study, 210 hospitalized psychiatric patients were selected by simple randomization from all records of hospitalized patients. The instrument of gathering data was a demographic checklist including age, gender, marital status, education, type of disorder and substance abuse and duration of psychiatric disorder. Data were analyzed by SPSS version 16 using Fisher exact and Chi square tests.

**Results:**

The mean age of patients was 37.9 years. Most of the patients were male, married and unemployed. The Prevalence of substance use was 36.7%. The most prevalent pattern of substance use was opium, opioid, methamphetamines and other substances (poly substance). The prevalence of substance use in patients with mood disorders was more than the other disorders and the most prevalent substance use in these patients was opium and opioid. Poly substance use was the most prevalent pattern of use (80 %) in psychotic and mood disorders due to substance. Significant difference was seen between genders, marital status, occupation, duration of illness and frequency of substance use (P < 0.05 ), however no significant difference was seen between educational levels, age and substance use.

**Conclusions:**

The patients with mood disorders had the highest comorbidity with substance use and concurrent use of poly substance was the most prevalent pattern of use in these patients. Therefore, successful treatment of psychiatric disorders and substance use needs multimodal and more serious interventions. Regarding to the pattern of poly substance use in these patients, careful screening should be performed at admission.

## 1. Background

The prevalence of patients with dual diagnosis (comorbidity) suffering from a psychiatric disorder along with co-occurring substance use disorder are increasing through time. Response to treatment in patients with this comorbidity is difficult, regarding the high rate of recurrence and non-compliance to the treatment (1). Substance use in the society has reached an endemic proportion and psychiatric wards are required to reflect the arising issues. The psychiatric wards population is disproportionately younger in age, male dominant and are mostly socially disorganized (2). The Patients with severe psychotic disorder use substance more than the general population. Substance use disorders in these patients have negative consequences, including recurrence of disorder, repeated admissions, homelessness and violence. Therefore, this comorbidity is of high importance regarding to the high rate of dependency and abuse of substance and its effects on course of psychiatric disorders (3). These patients compared with patients who are using drugs merely put more pressure on their families and society (4). Using drugs such as Lysergic acid diethylamide (LSD), cannabis and amphetamines produce symptoms like schizophrenia that make diagnosis difficult (5, 6). Many mental disorders are associated with an increased risk of later substance use conditions (7). Three types of relations are discussed between substance use and psychosis:

1. Substance use may lead to psychosis onset; therefore, either a cause or precipitant factor;
2. Substance use after onset of psychosis.
3. Substance use beginning concurrent with psychosis onset without affecting each other (8).

The systemic review study of Le Bec et al. showed that there was a relationship between cannabis and psychosis onset and cannabis consumption may be as an independent risk factor of psychotic disorders (9). Dequardo study on substance use pattern among patients with schizophrenia showed that 20% of females and 48% of males had substance abuse and the most common substance was cannabis (28%) following by alcohol (21%) (10). Goswami study on the course of substance use of 22 patients with schizophrenia showed that 11 patients used opioids, 9 patients consumed alcohol, 5 patients consumed cannabis and three patients consumed several kinds of drugs simultaneously (11). Regier et al. reported that at least 20% of patients with severe and chronic psychiatric disorders had substance abuse history and 50% of them had experienced substance use at least once during their lives. In this research, the prevalence of substance use in mood disorders, anxiety disorders, schizophrenia, personality disorders and other psychiatric disorders were 19.4%, 11.9%, 27.5%, 42%, and 14.7%, respectively (12). Baigent pointed out that there is a relationship between drug self-medication to alleviate anxiety and a higher risk of anxiety disorders and at first, therapists should principally be aware to detect substance use in patients with anxiety and mood disorders. Furthermore, he stated that there was a relationship between chronic substance use and certain personality disorders (13). Hosseini study on comorbidity of substance use and psychiatric disorders in outpatients showed that 12.7 % had current substance use and 3.3% had recent substance use. Opium was the most common substance and mood disorders had the most comorbidity with substance abuse (14). The study of Ghaleiha in Hamadan on the patients hospitalized in psychiatric wards reported that about half of them had comorbidity with substance abuse (15).

## 2. Objectives

Considering the difference in prevalence of psychiatric disorders comorbidity and substance use in different societies and races (16), harms of substance to individual and society, the necessity of timely diagnosis of comorbid disorders and lack of study on patterns of substance abuse among patients in psychiatric hospitals in Iran, this study assessed the patterns and comorbidity of substance use in hospitalized psychiatric patients. 

## 3. Patients and Methods

This is an analytical-cross sectional study. The statistical population of the research includes all the patients hospitalized in Kashan educational psychiatric hospital in 2013, Iran. The number of sample study was calculated 210 cases with consideration of CI = 95 %, P = 0.16 (14) and d = 0.05. We used simple randomization according to the table of random numbers. The samples were selected randomly from nearly one thousand records of hospitalized patients in the psychiatric wards of Kashan University of Medical Sciences in 2013. Inclusion criteria were age over 18 years old, admission in the year 2013 at the mentioned psychiatric hospital. Exclusion criteria were mental retardation, delirium, dementia, mood disorders and psychotic disorders due to other medical condition. 

### 3.1. Tools

Data collection tools consisted of a demographic checklist including age, gender, marital status, education, occupation, type of psychiatric disorders, type of substance and duration of disorder and substance use. Type of consuming substance was confirmed and determined by urine tests. Diagnostic records in this university hospital were written based on DSM-IV checklist. The Kappa Coefficient of this check list was 0.87. The checklist was provided by Noorbala et al. based on DSM-IV criteria (14). This structural questionnaire includes 149 symptoms of mental disorders such as mood disorder, anxiety, psychotic, psychosomatic, epilepsy, mental retardation, and organic mental disorders. The data were analyzed by using SPSS version16 (IBM, USA) using descriptive statistics, Fisher exact and Chi-square tests.

### 3.2. Ethical Consideration

The study protocol was approved by Kashan University of Medical Sciences (March 14th 2013 with Code 29/801/ 5434). These results were extracted from the authors’ MD degree thesis. Formal consent was obtained from hospital chief for using records. The records were used anonymously and all of the data were kept secret in this study.

## 4. Results

In this research, 210 patients’ records were evaluated. The mean age was 37.9 years. The most patients in the psychiatric wards were male, married and unemployed. The prevalence of substance use was 36.7% in these patients, which 18.1% of them had more than one psychiatric disorder except substance use disorder (Table 1).

**Table 1.** Demographic Characteristics of Hospitalized Psychiatric Patients^a^

Demographic Characteristics	Results, No. (%)
**Age, y**	
< 30	75 (27.1)
30 >	153 (72.9)
**Gender**	
Male	132 (62.9)
Female	78 (37.1)
**Education**	
Illiterate	16 (7.6)
Less high school	178 (84.8)
Over high school	16 (7.6)
**Current Substance Use**	
Yes	77 (36.7)
No	133 (63.3)

^a^ Data are presented as No. (%).

**Figure 1.**
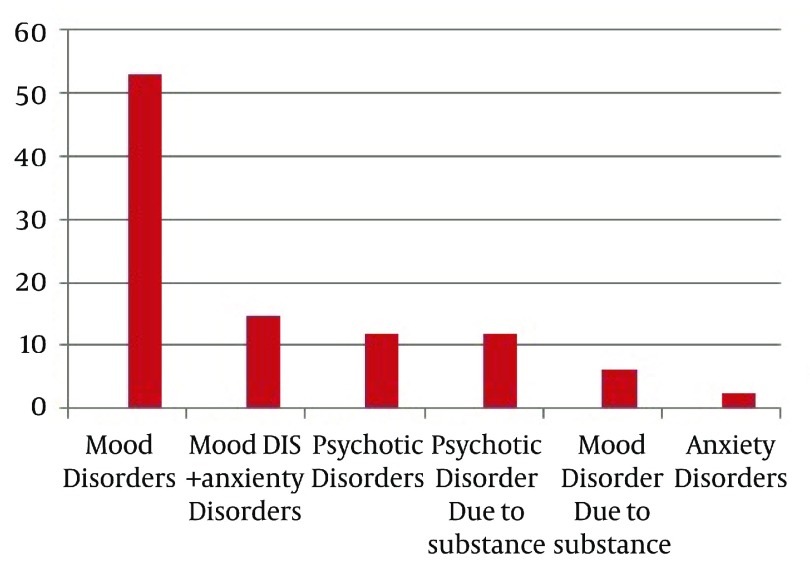
Frequency of Psychiatric Disorders in Hospitalized Patients

The most common psychiatric disorders among the hospitalized patients were mood disorders (52.9%) mood and anxiety disorders (14.7%), substance-induced psychotic disorder (11.9%) and psychotic disorders (11.9%), respectively (Figure 1).

**Figure 2.**
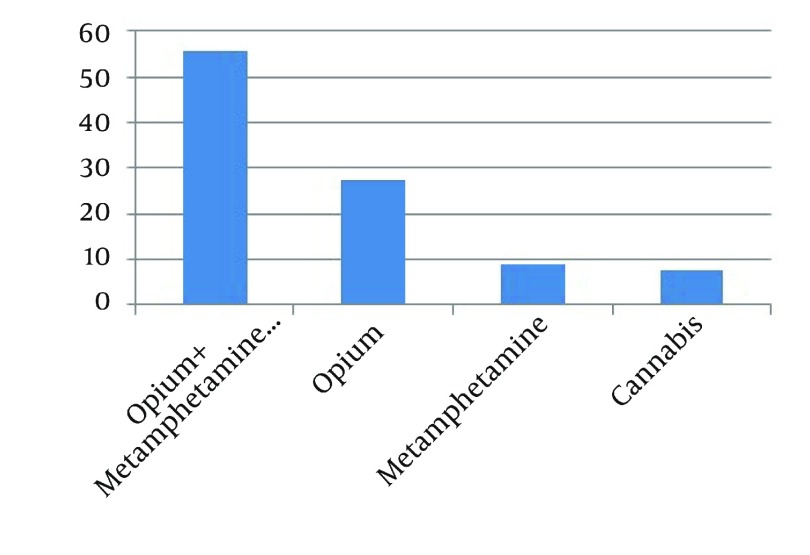
Frequency of Type of substance in Hospitalized Psychiatric Patients

Among patients who had substance abuse, 34 cases (44.2%) had used one kind of substance only, while 43 (55.8%) used more than one substance. Duration of substance use in 77.9% of patients was more than 5 years. Among those who were using substance less than 2 years, the most common substance was amphetamine compounds, while among those who were using substance for more than 2 years, poly substance use including opium, opioid and amphetamine compounds were more common. Overall, the most common pattern of substance use was opium with amphetamine and other substances (poly substances) in 55.8% patients (Figure 2).

The highest rate of substance use was among the patients with mood disorders (42.8%) comparing with other disorders. Opium and opioid was the most common substance among the patients with mood disorders, whereas, cannabis consumption was high among the patients with psychotic disorders. Consumption of several substances (poly substances) was the most common pattern of use among the patients with psychotic disorders due to substance and mood disorders due to substance (Table 2).

**Table 2.** Prevalence of Substance Use in Patients With Comorbidity of Substance Use Disorder Based on type of Psychiatric Disorders and Substance

Type of Substance	Mood Disorder and Anxiety Disorders	Psychotic Disorders	Psychotic Disorders Due to Substance	Mood Disorders Due to Substance	Total
**Opium and opioids**	15 (48.4)	3 (37.5)	3 (12)	0 (0)	21
**Metamphetamines**	4 (12.9)	0 (0)	2 (8)	1 (7.6)	7
**Methamphetamines opium, opioid, and other substance**	9 (29)	2 (25)	20 (80)	12 (92.4)	43
**Cannabis**	3 (9.7)	3 (37.5)	0 (0)	0 (0)	6
**Total**	31 (100)	8 (100)	25 (100)	13 (100)	77

Substance use rate among patients over 30-year-old and less than 30-year-old patients were 39.2% and 29.8%, respectively that this difference was not statistically significant. Female and male patients had a history of 11.5% and 51.5% substance use, respectively; significant statistically. By studying ‘marital status’, the divorced cases had the highest rate of substance use (66.7%) following by single patient, with significant difference (P < 0.05). The rate of substance use among cases with high school and secondary school degrees was 44.1% and the illiterate patients had lower substance use; but the difference was not statistically significant. Frequency of substance use among the unemployed patients (57%) was higher than the employed patients and the difference was also statistically significant (P = 0.0001). Frequency of substance use among the patients whose disorder duration was less than one year was higher (54.1%) than the patients whose duration of disorder was more than one year (P = 0.03) (Table 3).

In this study, significant difference was seen between gender, employment status, duration of disorder and marital status with substance use; whereas, statistically significant difference was not seen between age and educational level with substance use (Table 3).

**Table 3.** Frequency of Substance Use in Hospitalized Psychiatric Patients Based on Demographic Variables^a^

Substance Users Demographic Variables	Yes (n = 77)	No (n = 133)	Total (n = 210)	P Value
**Age, y**				0.20
< 30	17 (29.8)	40 (70.2)	57 (100)	
30 >	60 (39.2)	93 (60.8)	153 (100)	
**Gender**				0.0001
Female	9 (11.5)	69 (88.5)	78 (100)	
Male	68 (51.5)	64 (48.5)	132 (100)	
**Marital Status**				P < 0.05
Single	22 (44)	28 (56)	50 (100)	
Married	46 (31.9)	98 (68.1)	144 (100)	
Divorced	8 (66.7)	4 (33)	12 (100)	
Widow	1 (25)	3 (75)	4 (100)	
**Education**				P = 0.25
Illiterate	1 (6.3)	15 (93.8)	16 (100)	
Primary school	22 (36.7)	38 (63.3)	60 (100)	
Secondary and high school	52 (44.1)	66 (55.9)	118 (100)	
Over high school	2 (12.5)	14 (87.5)	16 (100)	
**Occupation**				P = 0.0001
Jobless	44 (57.1)	33 (42.9)	77 (100)	
Worker	6 (37.5)	10 (62.5)	16 (100)	
self-employed	10 (45.5)	12 (66.7)	22 (100)	
Staff	3 (45.5)	12 (54.5)	15 (100)	
Retired	4 (36.4)	7 (63.6)	11 (100)	
Housekeeper	8 (12.5)	56 (87.5)	64 (100)	
College student	0 (0)	5 (100)	5 (100)	
**Duration of Disorder, y**				P=0.003
< 1	33 (54.1)	28 (45.9)	61 (100)	
1-5	27 (32.5)	56 (67.5)	83 (100)	
5 >	17 (25.8)	49 (74.2)	66 (100)	

^a^ Data are presented as No. (%).

## 5. Discussion

This study was conducted to determine prevalence of comorbidity and pattern of substance abuse among hospitalized psychiatric patients in Kashan Psychiatric Hospital in 2013. The findings of the study showed that 36.7% of the patients had substance use comorbidity. The study of Ndetei in Kenya Psychiatric Hospital reported comorbidity of substance use and psychiatric disorders as 35%, which is almost consistent with the present study (17). whereas, the prevalence of substance use in the study of Katz (19) and the study of Rodriguez et al. (18) in Spain were 24% and 24.9%, respectively that these rates are lower than the rate of this study. Also, the rate of comorbidity of substance in the present study was higher than the study of Hosseini (14). The Prevalence of substance use in the studies of Hauli (21) in Tanzania among psychiatric patients, Sinclair (20) and Ghaleiha (15) in Hamadan were 68.5%, 74.8% and about 50%, respectively, which are higher than the rates obtained in the present study. The differences in these prevalence demonstrated could be due to socioeconomic, cultural and geographical differences (22). In this study, 55.8% of the patients who had substance use comorbidity used more than one type of substance. This rate is higher than Katz’s study in Israel, which 28.2%of psychiatric patients had used more than two substances (19). In the present study, the most common pattern of substance use was opium, opioid compounds and other substances, which is consistent with the study of Hosseini and Ghaleiha in Iran. In these two studies, the most common substance was also opium (14, 15). Whereas, in the study of Hauli in Tanzania, alcohol was the most prevalent substance use. These results were inconsistent with our findings (23). Among patients with mood disorders, opium and opioid were the most common substances, while cannabis used most common by the psychotic patients. The study of Dequardo showed that Cannabis was the most used substance among psychotic patients, which is consistent with our findings (10). Ringen revealed that schizophrenic patients mostly used stimulants and non-alcoholics substance, and bipolar patients mostly used alcohol (24), which are inconsistent with the results of this study. This difference might be due to the religious and cultural differences. In Islam religion, which is the official religion of Iran, drinking alcohol is prohibited and prosecuted. Therefore, access to alcoholic drinks is highly unlikely impossible for patients. The results indicated that 80% the patients with substance-induced psychosis was due to use of several substances (poly substance use) that no research was found regarding this issue for comparison, which may be due to lack of new studies on the effects caused by stimulants, such as amphetamines and combination of substances on psychotic patients. The rate of substance use co-morbidly in male psychiatric patients was significantly higher than females in this study, which was consistent with the studies of Hauli et al. (21), Mueser et al. (3), Katz et al. (19), Rodriguez et al. (18), Hosseini et al. (14), and Ghaleiha et al. (15) that male psychiatric patients have a higher risk of substance use. According to this study, the rate of substance use comorbidity among the unemployed patients was higher than the others, which are inconsistent with Ghaleiha study in psychiatric hospital of Hamadan (15). This difference could be due to differences in sampling method. In Ghaleiha study sampling method was convenient; while in our study, sampling method was simple randomization. Regardless of the fact that the patients were unemployed prior to developing psychiatric disorders or their psychiatric disorder precedes unemployment, it should be noted that unemployment is a predisposing factor for psychiatric disorders, delinquency and addiction. Unemployed persons have lower level of social responsibility, sense of worthlessness and they usually have tendency to use of substance. In the present study divorced patients and single patients had the most prevalence of substance use comorbidity respectively, which was consistent with the studies of Hosseini, Katz and Mericle (14, 19, 25), but inconsistent with the study of Hauli, which there was not significant relationship between marital status and substance use (21). Also in Behdani’s study on substance use in schizophrenic patients, there was no significant relationship between marital status and substance use (23). The findings also showed that the patients whose duration of disorder was less than 1 year had the highest frequency of substance use comorbidity. It may be explained that the patients have higher tendency to substance use in the acute phase of disorders. As the findings of the study showed, there was a high prevalence of substance use comorbidity among the patients hospitalized in psychiatric wards and the most common pattern of substance use was poly substance use. Therefore, it is proposed to identify substance use carefully and appropriately in the beginning of the admission and this procedure should be added to the routine screening of these patients. With considering these circumstances, further attentions should be paid to combined and multimodal treatments such as medications, counseling, family therapy and social rehabilitation in order to achieve better outcomes.

### 5.1. Limitation

This research was done in one region. In addition, sometimes substance screening test may be false negative because interaction between substance and other drugs. Furthermore, having inclusion and exclusion criteria may limit generalization of the results. However, information necessary to diagnose a substance use disorder may not always have been available due to the nature of the patients’ illnesses and thus limits the estimation of prevalence of these disorders.

### 5.2. Strong Points

This study has some strong point including detection of pattern and type of substance use among psychiatric inpatients which there is data limitation in this issue in recent years and in Iran no study find in this issue about pattern of sustance use in hospitalized patients at psychiatric wards. According to the research priorities in the field of substance use and recent epidemiological changes of substance including increase Metamphetamine, the present study addressed these topics.
